# The value of PRECISE-DAPT score and lesion complexity for predicting all-cause mortality in patients with NSTEMI

**DOI:** 10.1186/s43044-023-00329-6

**Published:** 2023-01-05

**Authors:** Gökhan Alıcı, Ömer Genç, Örsan Deniz Urgun, Tayfur Erdoğdu, Abdullah Yıldırım, Alaa Quisi, İbrahim Halil Kurt

**Affiliations:** 1 Department of Cardiology, Adana City Training and Research Hospital, Dr. Mithat Özsan Boulevard, 4522 Yuregir, Adana Turkey; 2 Department of Cardiology, Çam and Sakura City Hospital, Istanbul, Turkey; 3 Department of Cardiology, Çukurova State Hospital, Adana, Turkey; 4 Department of Cardiology, Adana Medline Hospital, Adana, Turkey

**Keywords:** Stroke risk scores, NSTEMI, PRECISE-DAPT score, SYNTAX score, In-hospital mortality

## Abstract

**Background:**

We aimed to evaluate the prognostic effects of stroke risk scores (SRS), SYNTAX score (SX score), and PRECISE-DAPT score on mortality in patients with non-ST-segment elevation myocardial infarction (NSTEMI). Three hundred forty-three patients hospitalized with a diagnosis of NSTEMI and underwent coronary angiography (CAG) between January 1, 2022, and June 1, 2022, were included retrospectively in this single-center study. Patients' demographic, clinical and routine biochemical parameters were recorded. The scores (CHADS_2_, CHA_2_DS_2_-VASc, R_2_CHA_2_DS_2_-VASc, ATRIA, SX score, PRECISE-DAPT) of each patient were calculated. Participants were then divided into two groups by in-hospital status; all-cause mortality (+) and all-cause mortality (−).

**Results:**

Overall, the mean age was 63.5 ± 11.8 years, of whom 63.3% (*n* = 217) were male. In-hospital mortality occurred in 31 (9.3%) patients. In the study population, those who died had significantly higher SX (*p* < 0.001), PRECISE-DAPT (*p* < 0.001), and ATRIA (*p* = 0.002) scores than those who survived. In logistic regression analysis, PRECISE-DAPT score [Odds ratio (OR) = 1.063, 95% CI 1.014–1.115; *p* = 0.012] and SX score [OR: 1.061, 95% CI 1.015–1.109, *p* = 0.009] were found to be independent predictors of in-hospital all-cause mortality among NSTEMI patients. In ROC analysis, the PRECISE-DAPT score performed better discriminative ability than the SX score in determining in-hospital mortality [Area under the curve = 0.706, 95% CI 0.597–0.814; *p* < 0.001].

**Conclusions:**

During the hospital stay, both PRECISE-DAPT and SX scores showed better performance than SRS in predicting all-cause mortality among NSTEMI patients undergoing CAG. Aside from their primary purpose, both scores might be useful in determining risk stratification for such patient populations.

## Background

The in-hospital prognosis and clinical consequences have become increasingly important as the frequency of acute myocardial infarction (AMI) has risen. Stroke risk scores (SRS), which are frequently used in clinical practice to predict stroke in patients with non-valvular atrial fibrillation, are scoring systems including risk factors similar to coronary artery disease (CAD) [1]. The relation of these scoring systems with mortality in patients with AMI has been shown in many studies [2, 3]. Although there are many thromboembolic risk scores, we only evaluated the four most commonly used ones (CHADS_2_, CHA_2_DS_2_-VASc, R_2_CHA_2_DS_2_-VASc, ATRIA) in our study.

PRECISE-DAPT score is another risk scheme developed to predict the risk of bleeding in patients treated with dual antiplatelet therapy after primary percutaneous coronary intervention. Only a few studies have focused on both in-hospital and long-term prognostic outcomes in AMI patients [4, 5]. SYNTAX score (SX score) is another scoring system that angiographically evaluates lesion complexity and severity of coronary artery tree. It has been also demonstrated that a high SX score is associated with poor clinical outcomes in patients with acute coronary syndromes [6, 7].

Although the effect of many risk factors on the development of mortality in NSTEMI patients has been proven, in this study, we aimed to overcome the lack of a study in the literature that examines and compares the effect of the PRECISE-DAPT, the SX score, and SRS on in-hospital all-cause mortality among patients with NSTEMI undergoing percutaneous coronary intervention.

## Methods

### Study population

This retrospective, single-center, cross-sectional and observational study was performed at the cardiology clinic of Adana City Training and Research Hospital between January 2022 and June 2022. The files of patients admitted to the coronary intensive care unit of the Department of Cardiology with a diagnosis of NSTEMI on the specified dates were retrospectively examined.

**Fig. 1:**
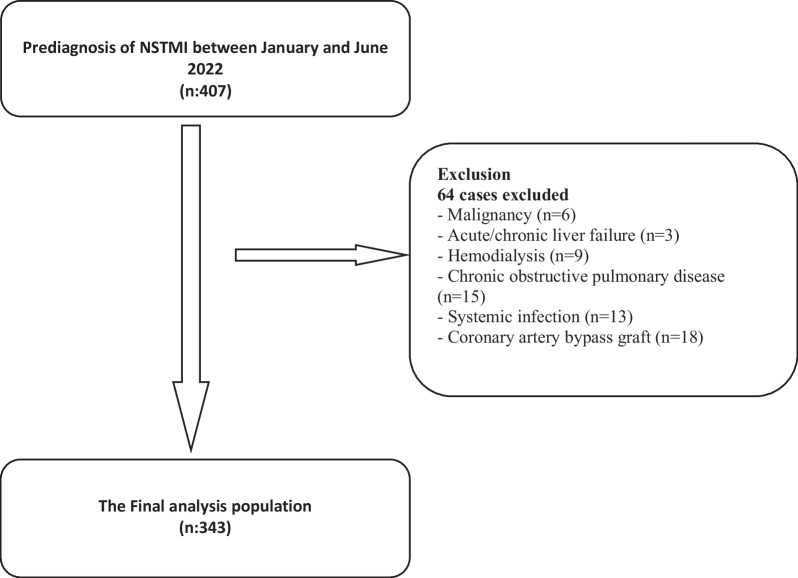
Flowchart of inclusion in the study

The diagnosis of NSTEMI has been made based on current clinical guidelines including positive cardiac markers including high-sensitivity cardiac troponin-I levels (hs-cTnT-I) (the upper limit of troponin-I in our laboratory was 0.16 ng/mL) without ST-segment elevation on routine electrocardiogram [8]. Clinical histories include hypertension (HT) and obstructive pulmonary disease, hyperlipidemia (HL), chronic renal disease (CKD), diabetes mellitus (DM), smoking, peripheral arterial disease, CAD, ischemic stroke/transient ischemic attack (TIA) were obtained and recorded in the national health record system, and medical files of each patient were also captured for retrospective evaluation. We excluded patients with coronary artery bypass graft, chronic obstructive pulmonary disease, severe liver and kidney disease, active cancer, and/or systemic infection (Fig. 1).

Routine blood samples were routinely obtained from all patients in the emergency triage unit or coronary intensive care unit. Laboratory analysis of routine venous blood samples was performed using an automated chemistry analyzer (Roche Diagnostic Modular Systems, Tokyo, Japan). The estimated glomerular filtration rate (eGFR) was measured using the Modification of Diet in Renal Disease formula.

### The definitions of scores

The PRECISE-DAPT score was determined for each patient using the web calculator (http://www.precisedaptscore.com). Unfortunately, we did not have data on the patients' previous bleeding history; therefore, this parameter could not be included when calculating the score.

CAG was performed on all subjects in our research. The femoral route was used for standard angiograms of the left and right coronary arteries to collect images. Coronary lesions with lumen diameter > 1.5 mm and at least 50% stenosis were analyzed separately, and the SX score was calculated using www.syntaxscore.com (version 2.10) [9]. The CHADS_2_ score consists of the following parameters; heart failure (HF) (1 point); HT (1 point); age ≥ 75 years (1 point); and history of stroke/TIA, or systemic embolism (2 points). The CHA_2_DS_2_-VASc score is calculated by assigning 2 points for age ≥ 75 years and history of stroke, TIA, or thromboembolism and 1 point for HF, HT, DM, vascular disease aged 65–75 years, and female gender [10]. The R_2_CHA_2_DS_2_-VASc score is determined by adding 2 points to the CHA_2_DS_2_-VASc score for renal failure defined as eGFR below 60 calculated from the Chronic Kidney Disease Epidemiology equation [2]. The ATRIA score assigns one point for the female sex, DM, HT, HF, proteinuria, and kidney dysfunction (eGFR < 45 mL/min/1.73 m2 or end-stage renal disease), and age classes (< 65, 65–74, 75–84, and ≥ 85 years) are assigned different scores based on stroke history (between 0 and 9) [11]. All scores were determined as previously described by two cardiologists blinded to patient survival data. The study population was divided into two groups by in-hospital mortality status: mortality (+) (*n* = 31) and mortality (−) (*n* = 312).

### Ethics approval and consent to participate

The study was carried out according to the recommendations set forth by the Declaration of Helsinki on biomedical research involving human subjects. The study was approved by the ethics committee of Adana City Training and Research Hospital (date: 08.08.2022, decision No: 2062). Due to the retrospective design, informed consent was not obtained by the ethics committee rules.

### Statistical analysis

Data analyses were performed using IBM SPSS Statistics for Windows, Version 22.0 software (IBM Corp., Armonk, NY, USA). Continuous variables with normal distribution have been expressed as mean ± standard deviation, whereas those with abnormal distribution have been expressed as median and interquartile range (IQR). Categorical variables have been expressed as numbers (*n*) and percentages (%). Student *t* test or Mann–Whitney *U*-test was used to compare continuous variables. The Chi-square test or Fisher’s exact test was used to compare categorical variables. The odds ratio (OR) and 95% confidence interval (CI) were calculated for each independent variable. Multivariate logistic regression analysis was performed to identify independent predictors of in-hospital all-cause mortality. Variables with significant *p* values (< 0.05) in univariate analysis were entered into multivariate analysis. The results of univariate and multivariate regression analyses were presented as OR with 95% CI. The area under the curve (AUC) and receiver operating characteristics (ROC) curve analyses were used to examine the prediction accuracy and performance of PRECISE-DAPT and SX scores for in-hospital mortality. Statistical significance has been defined as *p* < 0.05 throughout the study.

## Results

A total of 343 hospitalized NSTEMI patients [63.3% (*n* = 217) males, mean age = 63.5 ± 11.8 years] have been included in the analysis. In-hospital mortality occurred in 31 (9.3%) patients. The mortality (+) group was older (*p* = 0.003) and had a higher rate of DM (*p* = 0.016) than the mortality (−) group. When laboratory parameters were compared, serum hemoglobin (Hgb) (*p* < 0.001) and eGFR (*p* < 0.001) were lower in the mortality group, whereas hs-cTnT-I levels were higher [2.6 (12.1) vs. 0.7 (4.9), *p* = 0.004]. The SX score [20.6 ± 11.3 vs. 12.5 ± 7.5, *p* < 0.001], PRECISE-DAPT score [22.0 ± 14.5 vs. 12.0 ± 10.6, *p* < 0.001], and only the ATRIA risk score [4.5 ± 2.8 vs. 3.0 ± 2.3, *p* = 0.002] from SRS were statistically significantly higher in the non-survivors than in the survivors. Detailed demographic, clinical characteristics, and laboratory data of the participants are summarized in Table 1.

**Table 1:** Demographic and laboratory parameters of the study population

Variable	All (*n* = 343)	Non-survivor (*n* = 31)	Survivor (*n* = 312)	*p*-value*
Age (year), mean ± SD	63.5 ± 11.8	69.6 ± 13.2	62.9 ± 11.5	**0.003**
Male gender, *n* (%)	217 (63.3)	24 (77.4)	193 (61.9)	0.087
*Previous medical history*				
HT, *n* (%)	182 (53.1)	14 (45.2)	168 (53.8)	0.355
CAD *n* (%)	34 (9.9)	2 (6.5)	32 (10.3)	0.499
DM, *n* (%)	83 (24.2)	13 (41.9)	70 (22.4)	**0.016**
Previous stroke/TIA, *n* (%)	19 (5.5)	1 (3.2)	18 (5.8)	0.555
Smoking, *n* (%)	100 (29.2)	7 (22.6)	93 (29.8)	0.398
*Laboratory parameters*				
hs-CRP	0.5 (1.4)	1.9 (5.7)	0.5(1.0)	0.069
Hgb (g/dl), mean ± SD	12.9 ± 1.9	11.6 ± 1.8	13.1 ± 1.8	** < 0.001**
WBC (nmol/L), mean ± SD	11.2 ± 7.0	12.3 ± 5.5	11.1 ± 7.2	0.346
eGFR, mean ± SD	100.3 ± 36.7	77.9 ± 40.9	102.5 ± 35.6	** < 0.001**
hs-cTnT-I, pg/mL, median, [IQR]	0.74 (5.9)	2.6 (12.1)	0.7 (4.9)	**0.004**
Platelet count, × 10^3^/uL	264.2 ± 73.6	252.8 ± 77.6	265.7 ± 73.1	0.377
*Scores*				
CHADS_2_, mean ± SD	1.3 ± 1.0	1.6 ± 0.8	1.3 ± 1.0	0.109
CHA_2_DS_2_-VASc, mean ± SD	2.2 ± 1.5	2.5 ± 1.3	2.2 ± 1.5	0.283
ATRIA, mean ± SD	3.1 ± 2.4	4.5 ± 2.8	3.0 ± 2.3	**0.002**
R_2_CHA_2_DS_2_-VASc, mean ± SD	3.2 ± 1.8	3.7 ± 1.7	3.1 ± 1.7	0.073
SX, mean ± SD	13.3 ± 8.2	20.6 ± 11.3	12.5 ± 7.5	** < 0.001**
PRECISE-DAPT, mean ± SD	13.1 ± 11.4	22.0 ± 14.5	12.0 ± 10.6	** < 0.001**

*p value was calculated using an independent samples *t* test or the Mann–Whitney *U*-test for continuous variables and a chi-squared test or the Fisher’s exact test for categorical variables, as appropriate. *p value < 0.05 was considered significant

*Abbreviations*: CAD, coronary artery disease; DM, diabetes mellitus; hs-cTnT-I, high-sensitivity cardiac troponin-I; Hgb, hemoglobin; hs-CRP, high-sensitivity C-reactive protein; HT, hypertension; SX, SYNTAX score; WBC, white blood cell. Values are *n* (%), median (interquartile range [IQR]), or mean ± standard deviation

*p* value < 0.05 was considered significant

**Fig. 2:**
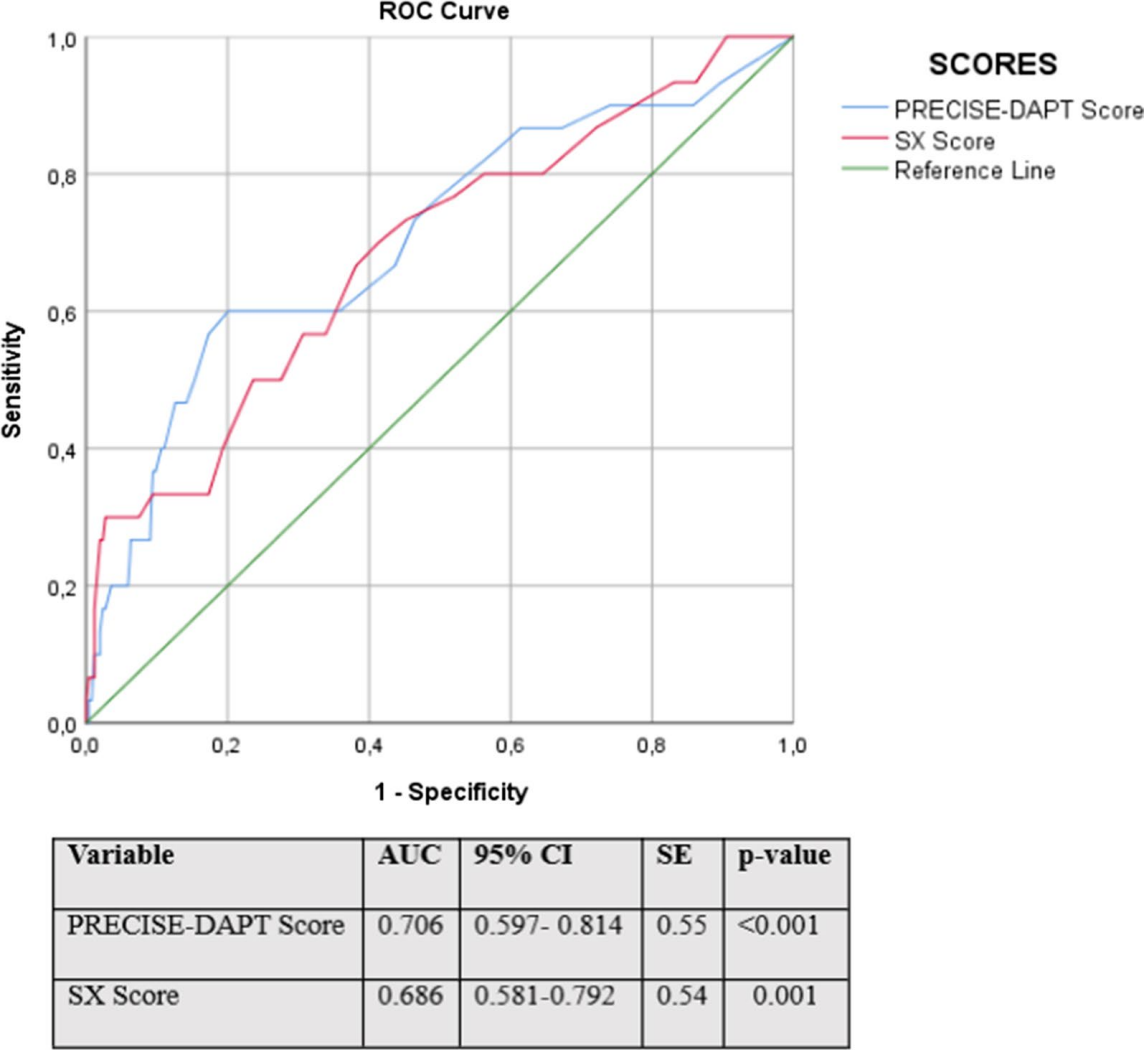
Receiver operating characteristic curve analysis of PRECISE-DAPT and SX scores for predicting in-hospital mortality

On multivariate logistic regression analysis, PRECISE-DAPT score (OR: 1.063, 95% CI 1.014–1.115, *p* = 0.012) and SX score (OR: 1.061, 95% CI 1.015–1.109, *p* = 0.009) were found to be independent predictors of in-hospital all-cause mortality among NSTEMI patients (Table 2). In ROC curve analysis, the PRECISE-DAPT score had a better discriminatory performance than the SX score in determining in-hospital all-cause mortality [AUC = 0.706, 95% CI 0.597–0.814, *p* < 0.001] (Fig. 2).

**Table 2:** Univariate and multivariate analysis of in-hospital mortality

Variable	Univariate analysis		Multivariate analysis	
	OR (95% CI)	*p* value	OR (95% CI)	*p* value
PRECISE-DAPT	1.064 (1.033–1.095)	** < 0.001**	1.063 (1.014–1.115)	**0.012**
ATRIA	1.072 (1.023–1.123)	**0.004**		
SX score	1.100 (1.058–1.145)	< **0.001**	1.061 (1.015–1.109)	**0.009**
hs-cTnT-I	1.000 (0.995–1.005)	0.977		

*Abbreviations*: hs-cTnT-I, high-sensitivity cardiac troponin-I; SXscore, SYNTAX score; CI, confidence interval; OR, odds ratio

A *p* value < 0.05 was considered significant

## Discussion

The main findings of the present study were as follows: (1) In-hospital mortality occurred in 31 (9.3%) patients with NSTEMI. Those who died were older and had a higher rate of DM. (2) No SRS had effectivity in determining the poor outcome during the hospital stay. PRECISE-DAPT and SX scores, on the other hand, were independent predictors of in-hospital all-cause mortality of NSTEMI patients.

Coronary artery disease-related deaths are among the most common causes of death worldwide. Of AMI patients, NSTEMI is approximately twice as common as ST-elevation myocardial infarction (STEMI) [12]. In-hospital mortality rates of patients with NSTEMI have been reported between 5.2 and 13.1% [13, 14]. The effect of many conditions on mortality has been described. Comorbidities such as multivessel disease, advanced age, low Hgb, atrial fibrillation (AF), CKD, diabetes, and history of HF have all been linked to a lower chance of survival in NSTEMI patients [15–17].

The PRECISE-DAPT score is determined by analyzing various values available from routine blood tests (Hgb, white blood cell count, and eGFR) and the risks associated with age. The utility of the PRECISE-DAPT score in contexts other than predicting bleeding risk has been investigated. For instance, it has been found that compelling evidence comparable to ours exists in two separate investigations on STEMI and NSTEMI patients that determine in-hospital mortality [5, 18]. It has also been researched to find out whether it has any effect on determining long-term prognosis in AMI patients [4].

Although the PRECISE-DAPT score was designed to assess bleeding risk in AMI patients, it was also found to be more predictive of mortality development than SRS in the present study. The influence of the PRECISE-DAPT score on many AMI complications other than mortality has also been studied in AMI patients. For example, the relationship with the development of periprocedural contrast-induced nephropathy [19], AF, and advanced atrioventricular block [20, 21] has been demonstrated in previous reports. Furthermore, the effect on the progression of the no-reflow phenomena in STEMI patients was identified [22]. Except for our study, there are no studies in the literature examining the relationship between PRECISE-DAPT and SRS, which are frequently used in routine cardiology practice, and mortality in NSTEMI patients.

The SX score has developed as a reproducible angiographic predictor for measuring the complexity of CAD based on coronary artery tree characteristics. In addition to the fact that the SX score simply represents the complexity of coronary artery lesions, extensive research has been conducted on its association with a variety of conditions in AMI patients [23–25]. We showed, similar to the existing literature, the SX score was found to be an independent predictor of mortality in AMI patients [26–29].

In our analysis, only the ATRIA score from SRS, which includes risk variables similar to CAD and has been used to predict the risk of thromboembolism in patients with non-valvular AF, was statistically higher in non-survivors than in survivors. However, the ATRIA score lost statistical significance in the multivariate regression analysis. Consequently, this is the first study to report that NSTEMI patients with high PRECISE-DAPT and SX scores had significantly higher in-hospital mortality than those with low scores, regardless of the prevalence of concomitant ischemic comorbidities.

## Limitations

The retrospective, single-center with a small sample size was the central limitation of the study. Unfortunately, we did not have information on the patient's previous history of bleeding, which is a significant factor in calculating the PRECISE-DAPT score. However, the fact that the strongest prediction among the scores included was in the PRECISE-DAPT score suggests that the result is underestimated at worst. As a result, the implications of the present study should be considered preliminary data. Prospective studies with larger populations should therefore be designed.

## Conclusions

This study showed that PRECISE-DAPT and SX scores have more robust evidence than SRS to predict mortality in NSTEMI patients. Thus, using the simple, practical, and widely used PRECISE-DAPT and SX scores not only determines the bleeding risk and extent of the coronary lesion, which have specific applications in the first medical contact, but it may also facilitate prognostic stratification for this patient group.

## Data Availability

The analyzed datasets are available from the corresponding author on request.

## Abbreviations

AF: Atrial fibrillation
AMI: Acute myocardial infarction
AUC: Area under the curve
CAD: Coronary artery disease
CAG: Coronary angiography
CKD: Chronic kidney disease
CI: Confidence interval
DM: Diabetes mellitus
eGFR: Estimated glomerular filtration rate
HF: Heart failure
Hgb: Hemoglobin
HL: Hyperlipidemia
HT: Hypertension
hs-cTnT-I: High-sensitivity cardiac troponin-I
IQR: Interquartile range
NSTEMI: Non-ST-segment elevation myocardial infarction
OR: Odds ratio
ROC: Receiver operating characteristics
SRS: Stroke risk scores
STEMI: ST-elevation myocardial infarction
SX score: SYNTAX score
TIA: Transient ischemic attack
